# Activation of the endocytosis pathway stratifies subtypes and therapeutic sensitivity in colorectal cancer

**DOI:** 10.1038/s41388-026-04019-w

**Published:** 2026-10-19

**Authors:** Hiroyuki Arai, Andrew Elliott, Yan Yang, Alex Farrell, Joshua Millstein, Fang-Shu Ou, Federico Innocenti, Jingyuan Wang, Francesca Battaglin, Priya Jayachandran, Sandra Algaze, Shivani Soni, Wu Zhang, Richard M. Goldberg, Michael J. Hall, Aaron James Scott, Jimmy J. Hwang, Emil Lou, Benjamin A. Weinberg, John Marshall, Sanjay Goel, Joanne Xiu, W. Michael Korn, Alan P. Venook, Heinz-Josef Lenz

**Affiliations:** 1 Division of Medical Oncology, Norris Comprehensive Cancer Center, Keck School of Medicine, University of Southern California, Los Angeles, CA USA; 2 Department of Clinical Oncology, St. Marianna University School of Medicine, Kawasaki, Japan; 3 Clinical & Translational Research, Medical Affairs, Caris Life Sciences, Phoenix, AZ USA; 4 Department of Population and Public Health Sciences, Norris Comprehensive Cancer Center, Keck School of Medicine, University of Southern California, Los Angeles, CA USA; 5 Alliance Statistics and Data Management Center, Mayo Clinic, Rochester, MN USA; 6 UNC Eshelman School of Pharmacy, University of North Carolina at Chapel Hill, Chapel Hill, NC USA; 7 West Virginia University Cancer Institute, Morgantown, WV USA; 8 Department of Clinical Genetics, Fox Chase Cancer Center, Philadelphia, PA USA; 9 Division of hematology/oncology, University of Arizona Cancer Center, Tucson, AZ USA; 10 Department of Solid Tumor Oncology, GI Medical Oncology, Levine Cancer Institute, Charlotte, NC USA; 11 Division of Hematology, Oncology and Transplantation, University of Minnesota, Minneapolis, MN USA; 12 Ruesch Center for the Cure of Gastrointestinal Cancers, Lombardi Comprehensive Cancer Center, Georgetown University, Washington, DC USA; 13 Department of Medical Oncology, Rutgers Cancer Institute, New Brunswick, NJ USA; 14 University of California, San Francisco, San Francisco, CA USA

**Keywords:** Colorectal cancer, Chemotherapy

## Abstract

Endocytosis regulates receptor trafficking and signaling, yet its role in colorectal cancer (CRC) remains unclear. We analyzed transcriptomic data from 15,025 CRC tumors to evaluate gene expression in clathrin-mediated endocytosis (CME) and the endosomal sorting complexes required for transport (ESCRT) pathway. Pathway-level signatures were compared across consensus molecular subtypes (CMS) and examined in relation to oncogenic signaling programs, while individual endocytosis-related genes were evaluated for associations with clinical outcomes in an independent randomized trial cohort. Expression of CME and ESCRT pathways varied by CMS, with the highest expression in CMS4 and the lowest in CMS3. Endocytosis signatures correlated with multiple oncogenic signaling pathways, including MAPK, TGF-β, WNT, PI3K, NOTCH, and angiogenesis, suggesting broad links between endocytic activity and tumor biology. In the randomized phase III CALGB/SWOG 80405 trial, low expression of *AP2M1*—a core adaptor in the CME complex—was significantly associated with longer overall survival and progression-free survival in patients treated with anti-EGFR agents, but not in those receiving anti-VEGF agents. These findings suggest that endocytic trafficking may be associated with oncogenic signaling and differential therapeutic benefit in patients with metastatic CRC. The endocytosis pathway reflects biologically relevant heterogeneity in CRC and may help guide therapeutic stratification.

## Introduction

Endocytosis is a fundamental cellular process that regulates the internalization, trafficking, recycling, and degradation of transmembrane proteins, including many key oncogenic receptors such as EGFR, HER2, FGFR, TGF-β, and Notch receptor families [1]. While traditionally viewed as a mechanism for signal attenuation, endocytosis also contributes to the spatial and temporal regulation of signal transduction, and under certain conditions, can even sustain oncogenic signaling through endosomal platforms [2–6]. In addition, components of the endocytic machinery have been implicated in the regulation of intracellular effectors, such as KRAS, highlighting their broader involvement in cancer-relating signaling networks [7].

The endocytic system is composed of distinct but interconnected pathways, including the clathrin-mediated endocytosis (CME) pathway, which facilitates selective internalization of membrane receptors and cargo via clathrin-coated vesicles, and the endosomal sorting complexes required for transport (ESCRT) pathway, which mediates cargo sorting within multivesicular bodies for degradation or recycling [8–12]. Both pathways are essential for receptor trafficking, membrane homeostasis, and intracellular signal control.

Recent proteogenomic studies have shown that endocytosis-related proteins are globally downregulated in colorectal cancer (CRC) cells compared to normal colonic epithelium [13]. However, it remains unclear how this downregulation affects tumor biology, especially given the dual role of endocytosis in both signal attenuation and maintenance. Notably, CRC is a highly heterogeneous disease composed of molecularly defined subtypes with distinct signaling architectures [14]. Yet, it remains unclear how endocytic pathways contribute to intracellular signaling networks across these subtypes, and whether their activity is altered in a uniform or subtype-specific manner.

Here, we conducted a large-scale transcriptomic and genomic analysis of over 15,000 CRC tumors from real-world patients to comprehensively characterize the activity of the endocytosis pathway and its associations with oncogenic signaling and immune-related modulators. We additionally explored the association between the endocytosis pathway activity and clinical outcomes in patients receiving standard first-line treatment in a randomized phase III clinical trial (CALGB/SWOG 80405). Our aim was to define the role of endocytosis in shaping CRC biology and to uncover associated molecular dependencies and generate hypotheses for potential therapeutic targeting.

## Methods

### Study population

This study included two distinct cohorts: a molecular profiling cohort and a clinical trial cohort. The molecular cohort consisted of formalin-fixed, paraffin-embedded (FFPE) tumor specimens collected from patients diagnosed with CRC, which were submitted to Caris Life Sciences (Phoenix, AZ), a laboratory certified under the Clinical Laboratory Improvement Amendments (CLIA). These cases were retrospectively reviewed for molecular characterization. The clinical cohort was derived from participants in the CALGB/SWOG 80405 trial (NCT00265850), a randomized phase III study that compared bevacizumab to cetuximab, each administered in combination with either FOLFOX or FOLFIRI, as first-line treatment for patients with metastatic CRC. CALGB is now part of the Alliance for Clinical Trials in Oncology. In the CALGB/SWOG 80405 trial, a total of 1137 patients with centrally confirmed *KRAS* exon 2 wild-type metastatic CRC were randomized to receive cetuximab- or bevacizumab-based chemotherapy (cetuximab arm: *n* = 578; bevacizumab arm: *n* = 559) [15]. For the current study, we analyzed RNA sequencing data from tumor tissue samples available in a subset of these patients. Specifically, 433 patients (cetuximab arm: *n* = 207; bevacizumab arm: *n* = 226) had sufficient quality and quantity of tumor RNA for whole-transcriptome sequencing (WTS) and were included in this study.

### Molecular profiling

In the Caris cohort, molecular characterization was performed on FFPE tumor tissues using both DNA- and RNA-based platforms. DNA was analyzed by next-generation sequencing (NGS) utilizing a 592-gene panel (Caris MI TumorSeek), and RNA underwent WTS. Details of sequencing procedures are provided in the Supplementary Methods. For variant interpretation, only those classified as “pathogenic” or “likely pathogenic” under the American College of Medical Genetics and Genomics (ACMG) guidelines were considered mutations, whereas alterations deemed “benign,” “likely benign,” or “variants of uncertain significance” were treated as wild type. The status of microsatellite instability (MSI) and DNA mismatch repair (MMR) was determined through a multimodal approach incorporating targeted NGS and immunohistochemistry, respectively, and tumors were categorized as either MSI-high (MSI-H)/deficient MMR (dMMR) or microsatellite stable (MSS)/proficient MMR (pMMR). PD-L1 expression was assessed by immunohistochemistry using the SP142 monoclonal antibody (Spring Biosciences). Membranous staining intensity in tumor cells was assessed using a four-tiered scale (0, 1 + , 2 + , 3 + ). PD-L1 positivity was defined as ≥5% of tumor cells showing moderate (2 + ) or strong (3 + ) staining. HER2 immunohistochemistry status was defined as positive if ≥10% of tumor cells exhibited strong (3 + ) staining, equivocal if ≥50% of tumor cells exhibited moderate (2 + ) staining, and negative for all other cases. The Caris consensus molecular subtype (CMS) classifier was developed using RNA sequencing data from the WTS platform (Supplementary Methods).

In the CALGB/SWOG 80405 trial cohort, RNA sequencing was conducted using the Illumina HiSeq 2500 system. Expression data were processed and normalized through the MAPRSeq version 3 computational pipeline [16].

### Statistical analysis

In the Caris cohort, to assess endocytic activity in CRC tumors, we generated composite gene expression signatures for CME (47 genes) and ESCRT (35 genes) pathways (Fig. S1), calculated as a sum of individual gene expression z-scores from TPM-normalized expression values (gene lists provided in Supplementary Methods). These composite scores were used to explore associations with clinical features and key molecular biomarkers. Correlative analyses between CME/ESCRT signatures and oncogenic signaling pathway activity were performed using pathway-specific gene sets (Supplementary Methods). For MAPK signaling, we used the MAPK Pathway Activity Score (MPAS), as previously described [17]. Spearman’s rank correlation coefficient was applied to evaluate associations between oncogenic signaling pathway scores and endocytosis activity. Correlation strength was interpreted as follows: coefficients <0.2 were considered negligible, 0.2–0.39 weak, 0.4–0.59 moderate, and ≥0.6 strong. Parallel analyses were conducted for individual genes within the endocytosis pathways.

In the Caris cohort, the expression levels of *CMTM6*, *CMTM4*, and *HIP1R* were analyzed in relation to immune-related features, including PD-L1 protein expression and MSI/MMR status. These genes were selected based on prior reports indicating their roles in the post-translational regulation of PD-L1. Specifically, CMTM6 and CMTM4 have been shown to stabilize PD-L1 at the cell surface by protecting it from lysosomal degradation, whereas HIP1R facilitates lysosomal targeting of PD-L1, thereby downregulating its expression [18–20].

In the CALGB/SWOG 80405 cohort, we investigated the association of 8 selected endocytosis-related genes (*CLTC*, *AP2M1*, *GAK*, *TSG101*, *VPS37A*, *PDCD6IP*, *VPS4A*, and *VPS4B*) with overall survival (OS) and progression-free survival (PFS) in patients receiving cetuximab- or bevacizumab-based first-line therapy. These 8 genes were selected because they represent core components of the endocytosis pathway and their essential regulators, encompassing both CME- (*CLTC*, *AP2M1*, and *GAK*) and ESCRT-mediated trafficking and degradation processes (*TSG101*, *VPS37A*, *PDCD6IP*, *VPS4A*, and *VPS4B*), and because their functional relevance to solid tumors including CRC has been previously reported [21–26]. OS was defined as the time from trial enrollment to death from any cause, and PFS was defined as the interval until radiologic progression or death. Gene expression levels were dichotomized into high and low groups based on median values. Multivariable Cox proportional hazards models were used to estimate hazard ratios for survival endpoints, adjusting for age, sex, race/ethnicity, ECOG performance status, tumor sidedness, number of metastatic sites, *RAS* and MSI status, and chemotherapy backbone regimen. Analyses were performed separately for each treatment arm.

Patients with missing data for any included variable were excluded from the relevant analyses. In the Caris cohort, where a large number of molecular features were analyzed, *p* values adjusted using the Benjamini–Hochberg method were calculated to account for multiple testing. In the CALGB/SWOG 80405 cohort, molecular data were limited to a smaller number of selected genes, multiple testing correction was not applied. All statistical tests were two-sided with a significance threshold of 0.05. Analyses were conducted using SPSS version 23 (IBM SPSS Statistics) and R version 4.1.0 (R Foundation for Statistical Computing, Vienna, Austria).

## Results

### Caris cohort: patient characteristics and genomic alterations in endocytosis-related genes

A total of 15,025 CRC samples were included in the molecular profiling cohort derived from the Caris dataset. The median patient age was 62 years. Left-sided tumors accounted for 64.1% of cases, and right-sided tumors for 35.9%. Samples were obtained from the primary tumor site in 56.6% and from metastatic sites in 43.4% (Table S1). No pathogenic or likely pathogenic mutations were detected in the 17 endocytosis-related genes included in the Caris MI TumorSeek panel. Gene amplifications were generally infrequent; the most commonly amplified gene was *ARFRP1* (1.03%, Table S2).

### Subtype-specific profiles in endocytosis pathways

**Fig. 1:**
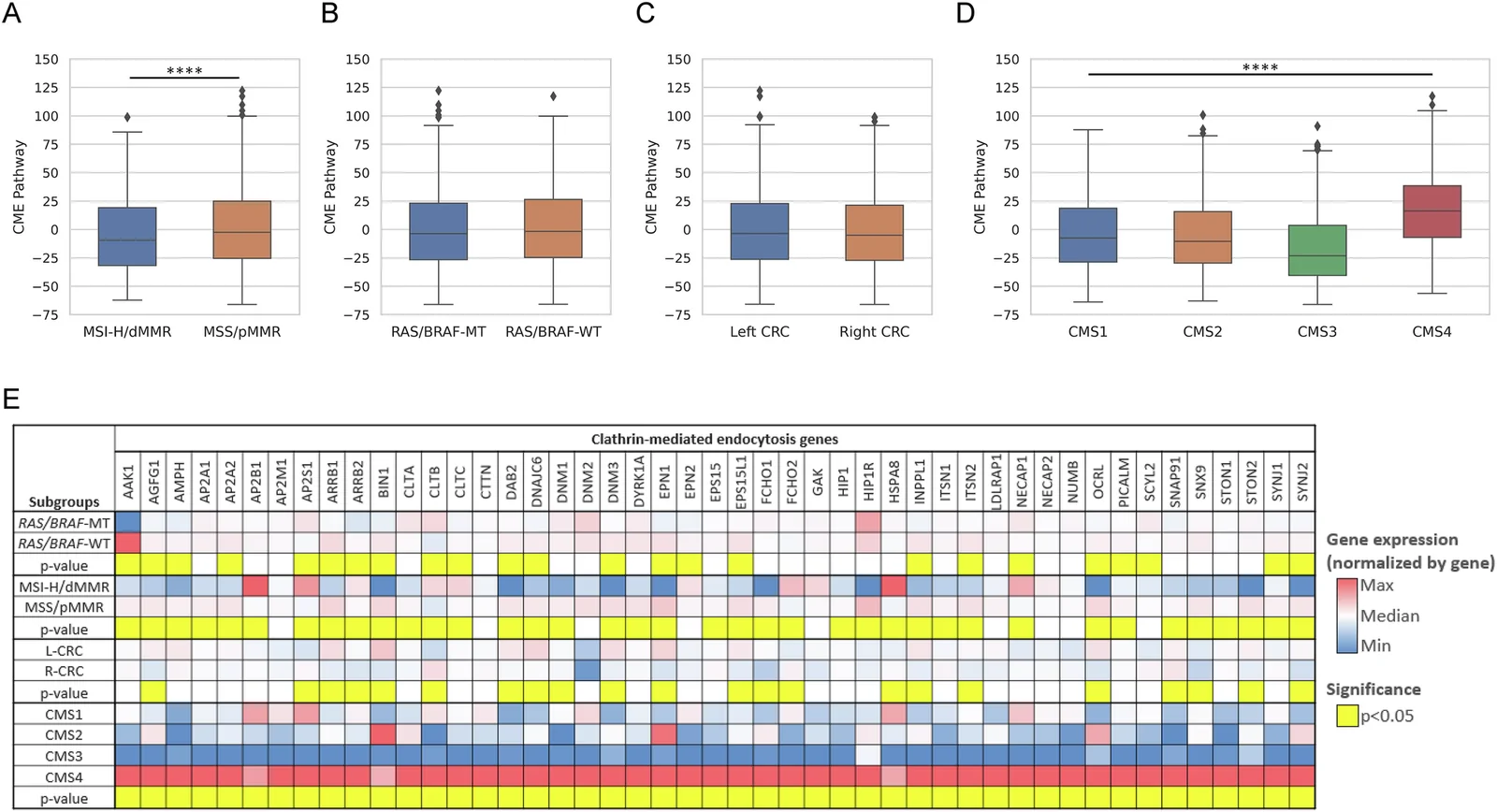
Association between the clathrin-mediated endocytosis (CME) pathway and clinical subgroups in colorectal cancer (CRC). Panels **A**–**D** display CME pathway activity (z-scores) across clinically defined subtypes: MSI status (**A**), *RAS/BRAF* mutation status (**B**), primary tumor location (**C**), and consensus molecular subtype (CMS) (**D**). An asterisk (****) indicates *p* values less than 0.0001. Panel **E** shows a heatmap of expression levels for 47 CME-related genes stratified by subgroups. Red indicates higher expression and blue indicates lower expression. The heatmap reveals marked upregulation of CME-related genes in CMS4 tumors and globally reduced expression in CMS3 tumors. MT mutant, WT wild type.

CME pathway activity was examined across subgroups defined by *RAS*/*BRAF* mutation status, MSI/MMR status, tumor sidedness, and CMS. Median CME z-scores were significantly higher in MSS/pMMR tumors compared to MSI-H/dMMR (0.33 vs –5.69, *p* < 0.0001) tumors (Fig. 1A). No significant differences were observed by *RAS*/*BRAF* status or primary tumor location (Fig. 1B, C). Notably, CME activity was highest in CMS4 tumors and lowest in CMS3 (median z-scores: CMS1 = -3.76, CMS2 = –5.81, CMS3 = –16.45, CMS4 = 15.81; *p* < 0.0001) (Fig. 1D). Heatmap analysis showed that nearly all CME genes were upregulated in CMS4 and broadly downregulated in CMS3 (Fig. 1E).

**Fig. 2:**
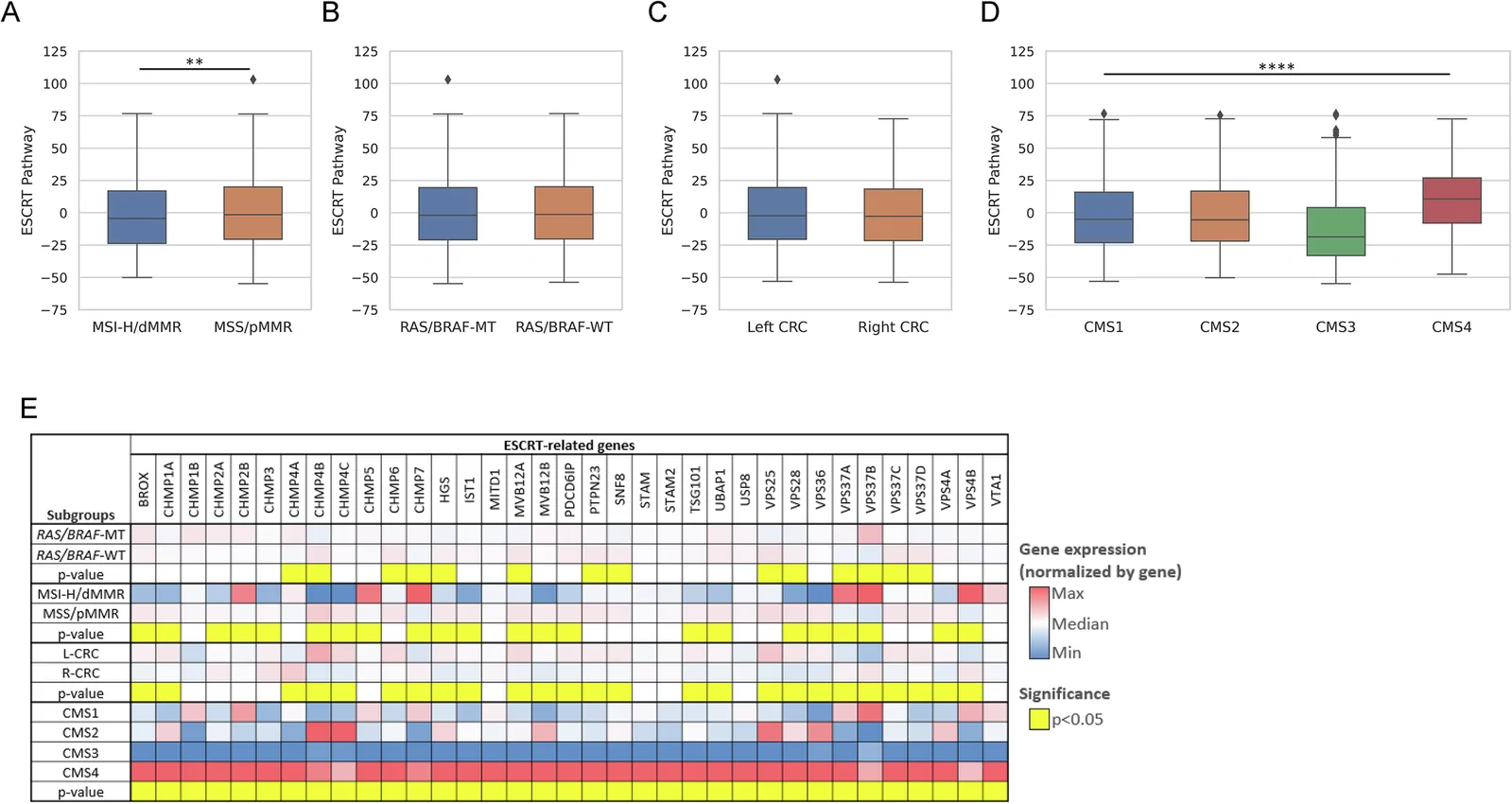
Association between the endosomal sorting complexes required for transport (ESCRT) pathway and clinical subgroups in colorectal cancer (CRC). Panels **A**–**D** display CME pathway activity (z-scores) across clinically defined subtypes: MSI status (**A**), *RAS/BRAF* mutation status (**B**), primary tumor location (**C**), and consensus molecular subtype (CMS) (**D**). An asterisk (****) indicates *p* values < 0.0001, and (**) indicates *p* values < 0.01. Panel **E** shows a heatmap of expression levels for 35 ESCRT-related genes stratified by subgroups. Red indicates higher expression and blue indicates lower expression. The heatmap reveals marked upregulation of ESCRT-related genes in CMS4 tumors and globally reduced expression in CMS3 tumors. MT mutant, WT wild type.

Similar trends were observed for the ESCRT pathway. ESCRT z-scores were significantly higher in MSS/pMMR tumors compared to MSI-H/dMMR (median, 0.18 vs –2.55; *p* < 0.01), with no differences by *RAS*/*BRAF* status or sidedness (Fig. 2A–C). Again, pathway activity was highest in CMS4 and lowest in CMS3 (median z-scores: CMS1 = -2.57, CMS2 = -1.86, CMS3 = –12.85, CMS4 = 9.56; *p* < 0.0001) (Fig. 2D), with heatmaps revealing robust expression of ESCRT genes in CMS4 and reduced expression in CMS3 (Fig. 2E).

### Association between endocytosis and oncogenic signaling pathways

**Fig. 3:**
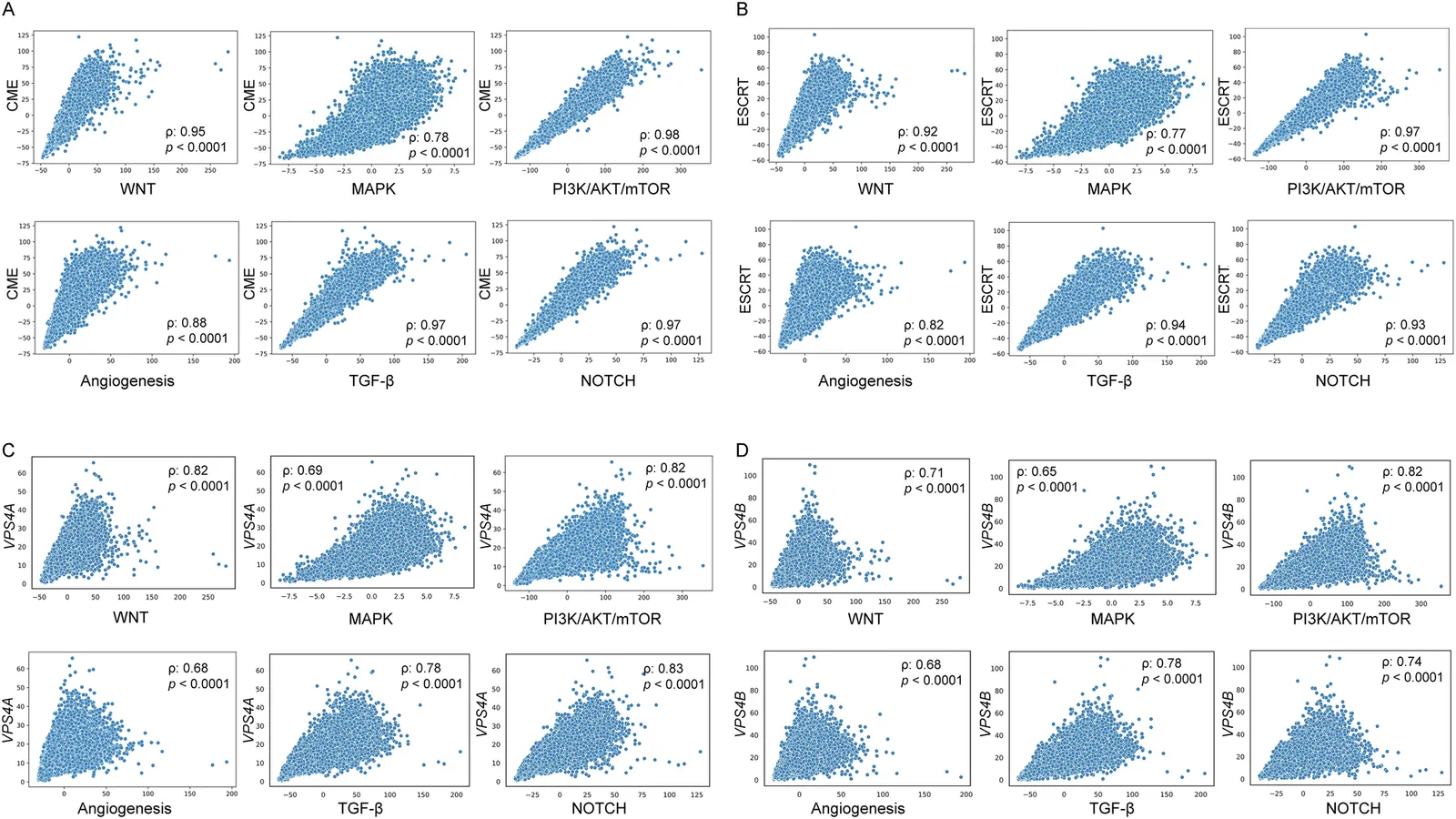
Correlation between endocytosis pathway activity and key oncogenic signaling pathways in colorectal cancer. Panel **A** shows correlations between the clathrin-mediated endocytosis (CME) pathway and the activity of six major oncogenic signaling pathways (WNT, MAPK, PI3K/AKT/mTOR, angiogenesis, TGF-β, and NOTCH), while panel **B** displays the corresponding analysis for the endosomal sorting complexes required for transport (ESCRT) pathway. Panels **C** and **D** show associations between *VPS4A* and *VPS4B* gene expression levels and the same oncogenic pathway signatures. All pathway activities were evaluated using z-score–based analyses. Strong positive correlations were observed between endocytosis-related signatures/genes and each of the six oncogenic signaling pathways across all analyses.

CME and ESCRT pathway activities were strongly correlated (ρ = 0.98, *p* < 0.0001) (Fig. S2). CME signature scores were strongly and positively correlated with all six major oncogenic signaling pathways—WNT, MAPK, PI3K/AKT/mTOR, angiogenesis, TGF-β, and NOTCH (*ρ* = 0.78–0.98, all *p* < 0.0001) (Fig. 3A). ESCRT signatures showed similar positive correlations (*ρ* = 0.77–0.97, all *p* < 0.0001) (Fig. 3B). Expression levels of *VPS4A* and *VPS4B*, genes encoding core ESCRT components, were also strongly and positively correlated with all six pathway scores. For *VPS4A*, ρ ranged from 0.68 to 0.83 (all *p* < 0.0001), and for *VPS4B*, ρ ranged from 0.65 to 0.82 (all *p* < 0.0001) (Fig. 3C, D).

### Association between endocytosis pathways and HER2 protein expression

**Fig. 4:**
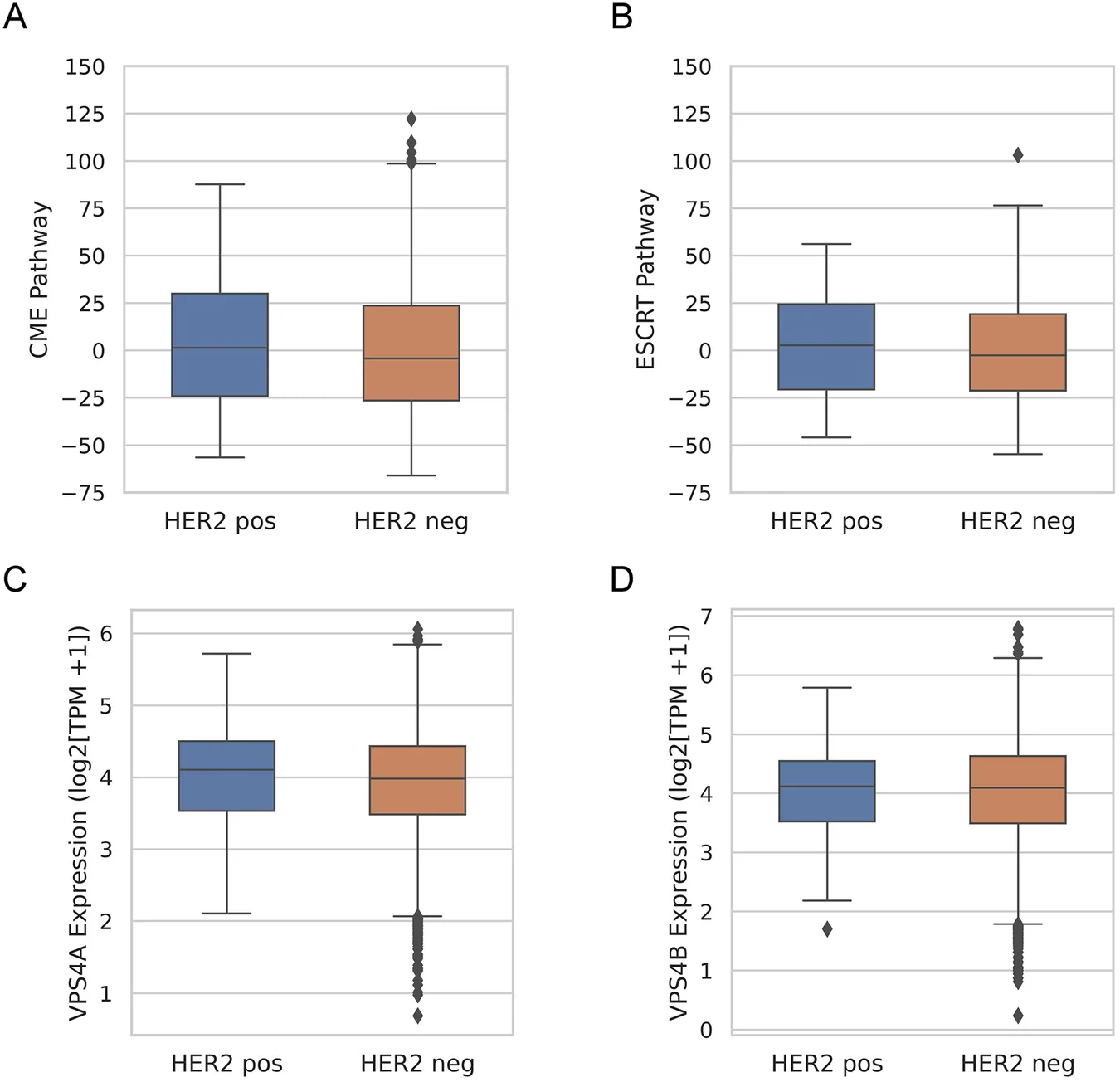
Association between endocytosis pathway activity and HER2 protein expression status in colorectal cancer. Panels **A** and **B** show z-score–based expression of the clathrin-mediated endocytosis (CME) and the endosomal sorting complexes required for transport (ESCRT) pathways, respectively. Panels **C** and **D** display individual expression levels of *VPS4A* and *VPS4B*. In all panels, the x-axis represents HER2 status, comparing HER2–positive (HER2 pos) and HER2–negative (HER2 neg) tumors. No significant differences were observed in pathway activity or gene expression levels between HER2 subgroups in any of the analyses.

CME and ESCRT pathway scores did not significantly differ between HER2–positive and HER2–negative tumors (median CME z-scores: 1.31 vs –4.23, *p* = 0.12; median ESCRT z-scores: 2.66 vs –2.67, *p* = 0.20) (Fig. 4A–B). Likewise, *VPS4A* and *VPS4B* expression levels were not associated with HER2 status (median *VPS4A* z-scores: 4.11 vs 4.00, *p* = 0.35; median *VPS4B* z-scores: 4.19 vs 4.25, *p* = 0.69) (Fig. 4C, D).

### Association between endocytosis-related genes and immune-related markers

**Fig. 5:**
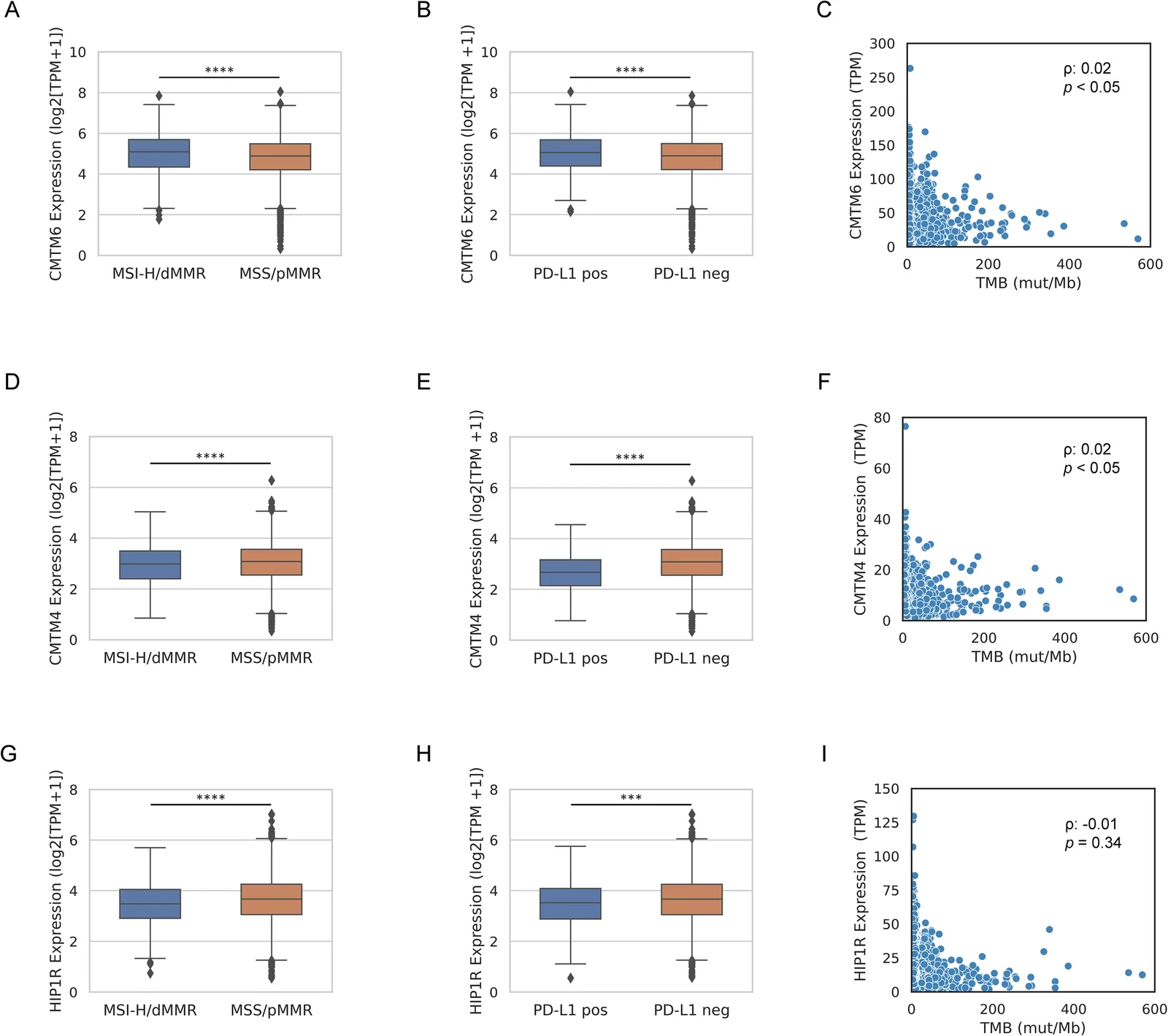
Association between the expression of endocytosis-related genes implicated in PD-L1 regulation and immune-related markers in colorectal cancer. Panels **A**–**C** show *CMTM6* expression levels, **D**–**F** show *CMTM4*, and **G**–**I** show *HIP1R*, in relation to MSI status (**A**, **D**, **G**), PD-L1 expression (**B**, **E**, **H**), and tumor mutational burden (TMB; **C**, **F**, **I**). An asterisk (****) indicates *p* values < 0.0001, and (***) indicates *p* values < 0.001. *CMTM6* expression was higher in MSI-H/dMMR and PD-L1–positive tumors, whereas *CMTM4* and *HIP1R* were more highly expressed in MSS/pMMR and PD-L1–negative tumors. None of the three genes showed meaningful correlation with TMB.

*CMTM6* and *CMTM4* expression levels were strongly correlated (ρ = 0.62, *p* < 0.0001) (Fig. S3). *CMTM6* expression was elevated in MSI-H/dMMR tumors compared to MSS/pMMR (median z-scores, 37.06 vs 32.68, *p* < 0.0001), and in PD-L1–positive tumors compared to PD-L1–negative tumors (median z-scores, 37.92 vs 32.77, *p* < 0.0001) (Fig. 5A–B). In contrast, *CMTM4* expression was lower in MSI-H/dMMR tumors compared to MSS/pMMR tumors (median z-scores, 7.83 vs 8.38, *p* < 0.0001) and in PD-L1–positive tumors compared to PD-L1–negative tumors (median z-scores, 6.19 vs 8.43, *p* < 0.0001) (Fig. 5D, E). Similarly, *HIP1R* expression was lower in MSI-H/dMMR tumors compared to MSS/pMMR tumors (median z-scores, 11.99 vs 13.95, *p* < 0.0001) and in PD-L1–positive tumors compared to PD-L1–negative tumors (median z-score, 12.60 vs 13.85, *p* < 0.001) (Fig. 5G, H). None of these genes (*CMTM6*, *CMTM4*, *HIP1R*) showed a meaningful correlation with tumor mutational burden (Fig. 5C, F, I).

### CALGB/SWOG 80405 cohort: association between endocytosis-related genes and clinical outcomes

**Fig. 6:**
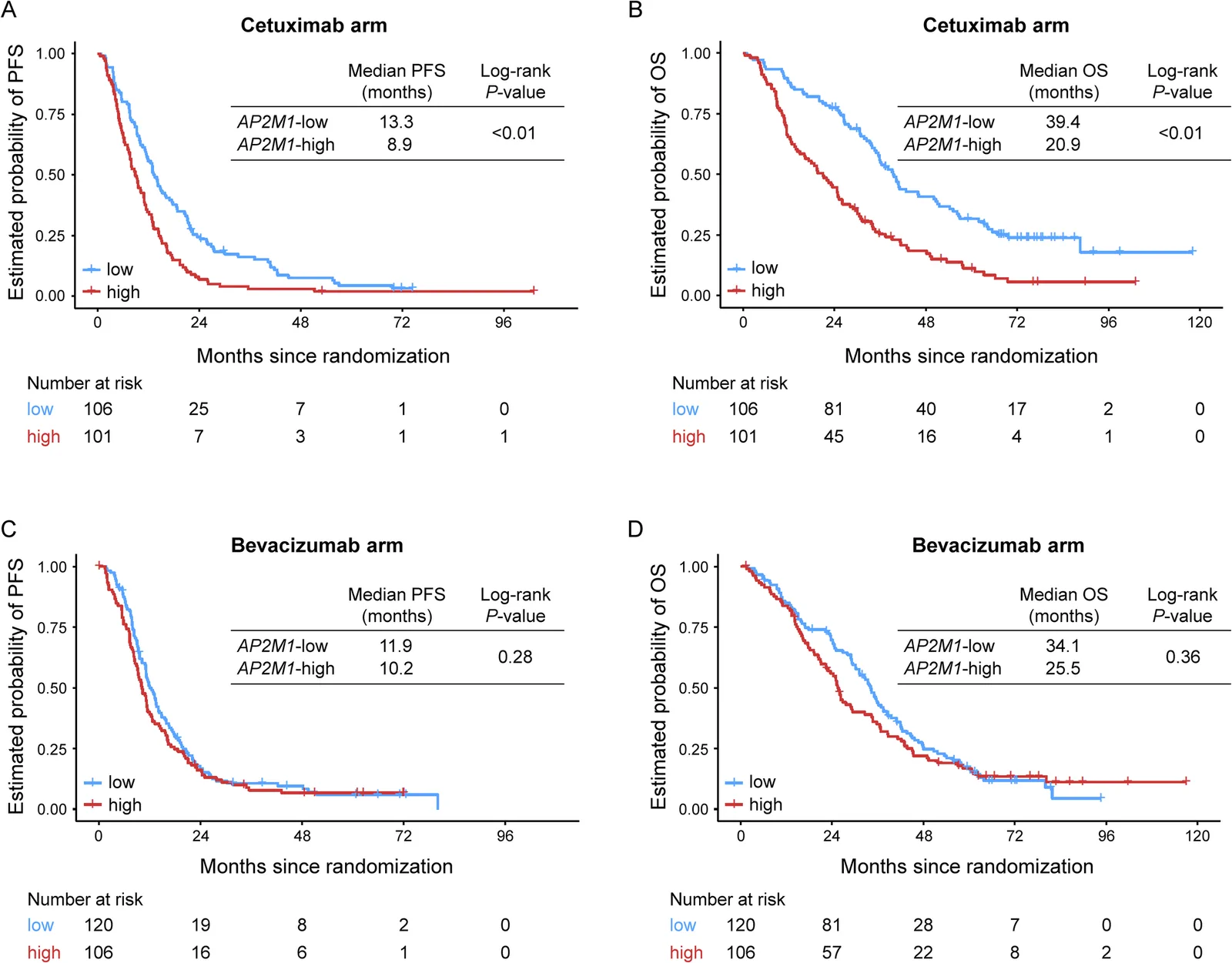
Association between *AP2M1* expression and clinical outcomes by treatment arm in the CALGB/SWOG 80405 trial. Kaplan–Meier survival analyses were conducted separately for cetuximab-based (**A**, **B**) and bevacizumab-based (**C**, **D**) regimens. Panels **A** and **B** show progression-free survival (PFS) and overall survival (OS), respectively, in patients receiving cetuximab. Panels **C** and **D** show PFS and OS, respectively, in patients treated with bevacizumab. In the cetuximab arm, patients with low *AP2M1* expression experienced significantly longer PFS and OS compared to those with high expression. In contrast, *AP2M1* expression level was not associated with survival outcomes in the bevacizumab arm.

In the CALGB/SWOG 80405 cohort, 433 patients (207 receiving cetuximab and 226 receiving bevacizumab) were analyzed. Baseline characteristics are shown in Table S3. Among the 8 selected endocytosis-related genes, *AP2M1* expression was significantly associated with clinical outcomes in univariable analysis (Table S4–S5). In the cetuximab-treated group, low *AP2M1* expression was associated with prolonged PFS (median 13.3 vs. 8.9 months, log-rank *p* < 0.01) and OS (median 39.4 vs. 20.9 months, log-rank *p* < 0.01) compared to high expression (Fig. 6A, B). These associations remained significant after multivariable adjustment. No survival difference by *AP2M1* expression was observed in patients receiving bevacizumab (Fig. 6C, D). Significant interactions were observed between *AP2M1* expression and treatment group for both PFS and OS (*p* < 0.01). In contrast, *CLTC* expression was associated with OS only in the multivariable analysis in the cetuximab group, but no significant interaction was observed between *CLTC* expression and treatment arm (Tables S4–S5).

To further explore *AP2M1*’s biological relevance, we assessed its expression in the Caris cohort. *AP2M1* expression showed strong positive correlations with all six oncogenic pathway signatures (Fig. S4), and these associations persisted in *RAS*/*BRAF* wild-type tumors, particularly with the MAPK pathway (Fig. S5).

## Discussion

This study provides novel insights into the relationships between endocytosis pathways and CRC biology by integrating molecular defined subgroups, immune modulation, and therapeutic outcomes. To our knowledge, this is the first comprehensive analysis to link large-scale endocytic gene expression profiles with CRC molecular subtypes, oncogenic signaling, and treatment response, using both a real-world cohort of over 15,000 tumors and an independent randomized phase III clinical trial dataset. Our analysis combining multi-omic molecular data with clinical outcomes demonstrated that endocytosis pathway activity is closely associated with molecular features, oncogenic signaling, and therapeutic outcomes in CRC.

The distinct expression patterns of CME and ESCRT pathway genes across CMS highlight the biological specificity of endocytic activity. CMS4 tumors, which are defined by strong TGF-β signaling, stromal infiltration, and angiogenesis, showed the highest expression of endocytosis signatures [14]. This suggests that the high endocytic pathway activity observed in CMS4 may reflect the coordinated activation of intracellular trafficking and signaling programs characteristic of this subtype. In contrast, CMS3 tumors, driven primarily by metabolic reprogramming and often harboring *KRAS* mutations, exhibited the weakest association with endocytosis gene expression, suggesting reduced dependence on receptor-mediated signaling dynamics. This may reflect the biology of *KRAS*-mutant tumors, in which constitutive downstream activation of the MAPK and PI3K pathways can occur independently of receptor trafficking. However, the present analysis cannot determine whether this accounts for the lower endocytosis-related gene expression observed in CMS3 tumors.

Expression of genes in the CME and ESCRT pathways showed strong correlations with multiple oncogenic signaling pathways, including MAPK, WNT, TGF-β, and PI3K. Notably, CME and ESCRT pathway scores were highly correlated (ρ = 0.98), suggesting coordinated transcriptional activity in CRC. Despite this correlation, the two pathways represent biologically distinct sequential steps of endocytic trafficking rather than functionally redundant processes. These findings are particularly intriguing in light of previous reports that described global downregulation of endocytosis-related proteins in CRC cells compared to normal colonic epithelium, suggesting that the pathway may not play a conventional oncogenic driver role [13]. However, our data reveal that specific components of the endocytic machinery—notably those involved in late-stage trafficking such as VPS4A and VPS4B—are selectively retained or even upregulated in certain tumor subtypes such as CMS4, where they closely track with oncogenic pathway activation. This discrepancy suggests that, despite previously reported global reductions in endocytosis-related proteins, selected components of the endocytic machinery may remain highly expressed in specific CRC subtypes and may be coordinately associated with oncogenic signaling activity. These subtype-specific patterns raise the possibility that endocytic trafficking may have distinct biological relevance across CRC subtypes and warrant further functional investigation.

We also identified *AP2M1*, a core component of the CME complex, as a gene of potential biological and clinical interest. Its expression positively correlated with MAPK pathway activity. Paradoxically, however, low *AP2M1* expression was associated with improved clinical outcomes in cetuximab-treated patients. This apparent contradiction reflects the dual role of EGFR endocytosis. AP2M1 is essential for clathrin-coated pit formation and facilitates EGFR internalization [27]. While internalization can downregulate receptors, it may also sustain signaling through receptor recycling and endosomal signaling—mechanisms known to maintain MAPK activity [3]. One possible explanation for this observation relates to the established role of AP2M1 in clathrin-mediated EGFR internalization. Altered AP2M1 expression could theoretically influence the balance between cell-surface EGFR availability, receptor recycling, and endosomal signaling. However, the present study did not directly evaluate EGFR trafficking or membrane receptor abundance, and therefore this mechanistic interpretation remains hypothetical. These observations raise the possibility that EGFR pathway activity and sensitivity to anti-EGFR antibodies may not necessarily be captured by the same biological features. Accordingly, *AP2M1* warrants further investigation as a candidate biomarker of anti-EGFR treatment outcome, although its biological mechanism and predictive utility require independent validation.

Although we explored transcript-level expression of immune-related components such as CMTM6, CMTM4, and HIP1R, which have been implicated in PD-L1 recycling [28], their associations with PD-L1 expression and MSI status were weak and inconsistent. This indicates that while endocytosis may play a role in shaping the immune microenvironment, its contribution in CRC appears limited and warrants further mechanistic investigation.

Our study has several limitations. The analyses were primarily correlative and based on transcriptomic data. Therefore, the observed associations do not establish whether altered endocytic pathway activity is a cause or consequence of oncogenic signaling, or whether both reflect coordinated regulation by other biological processes. Functional validation, such as protein-level assessments or in vivo modeling, will be critical to confirm mechanistic hypotheses. In addition, because CMS subtypes differ in clinicopathologic and molecular characteristics, residual confounding by factors such as tumor sidedness and sample origin cannot be excluded when interpreting subtype-specific differences in endocytic pathway activity. Moreover, although low *AP2M1* expression was associated with improved outcomes in cetuximab-treated patients, the expression cutoff used in this analysis has not been clinically validated, and the finding has not been independently validated in an external anti-EGFR-treated cohort. Therefore, *AP2M1* should currently be regarded as a candidate predictive biomarker requiring further validation before clinical application.

Nevertheless, our findings provide a foundational framework for understanding how endocytosis-related transcriptional programs are associated with CRC molecular subtypes, oncogenic signaling, and therapeutic outcomes. They also highlight the need to move beyond canonical genetic alterations and consider intracellular trafficking dynamics in the design of future precision oncology strategies. The preferential upregulation of endocytosis pathway genes in CMS4 tumors may reflect coordinated transcriptional regulation of receptor trafficking and signaling programs characteristic of this subtype. Whether these subtype-specific endocytic patterns represent therapeutically actionable dependencies remains unknown and will require dedicated functional and preclinical studies.

Together, our findings shed new light on the understanding of the endocytosis pathway in CRC biology. By integrating transcriptomic and clinical outcome data, we identify strong associations between endocytic components, oncogenic signaling activity, and therapeutic outcomes. These observations provide a rationale for further investigation of intracellular trafficking as a potential biomarker and therapeutic context in CRC.

## Supplementary information

Figure S1

Figure S2

Figure S3

Figure S4

Figure S5

Supplementary Methods

Table S1

Table S2

Table S3

Table S4

Table S5

## Data Availability

De-identified datasets analyzed in the current study are available from the corresponding author on reasonable request.
