# Supplementary material for: Activation of the endocytosis pathway stratifies subtypes and therapeutic sensitivity in colorectal cancer
Source: Oncogene. 2026 Oct 19;45(46):5048–56. doi: 10.1038/s41388-026-04019-w (PMC13723413; doi:10.1038/s41388-026-04019-w)
Supplement: Supplementary file 1 [file 41388_2026_4019_MOESM1_ESM.pdf]

Figure S1

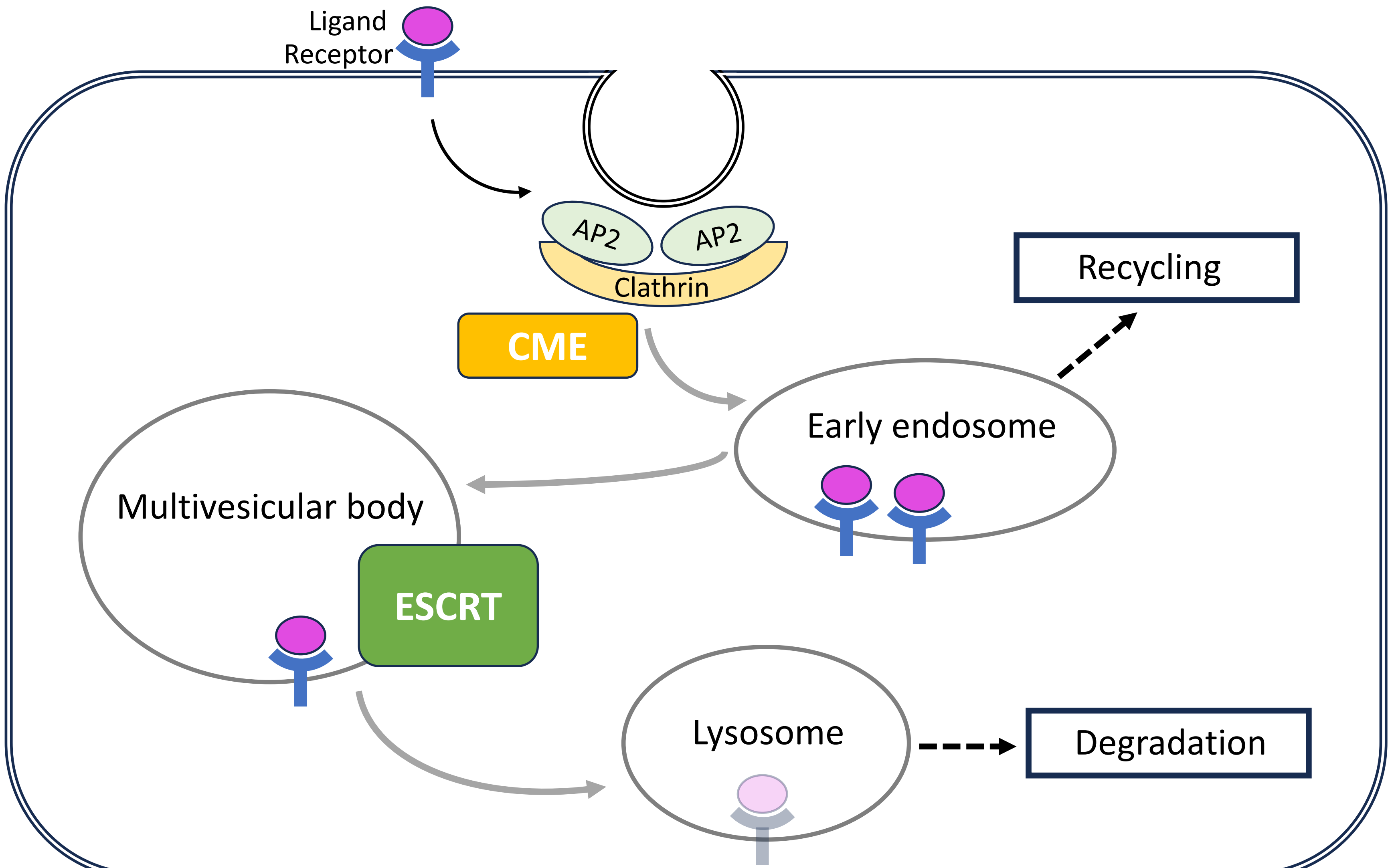

Ligand-bound receptors at the plasma membrane are internalized through clathrin-mediated endocytosis (CME) pathway, in which the AP2 adaptor complex and clathrin form a coated pit that buds into the cell. The internalized cargo enters the early endosome, which serves as a central sorting hub. From there, receptors can either be recycled back to the plasma membrane (recycling pathway) or targeted for degradation (degradation pathway). Degradation proceeds through the formation of a multivesicular body, where the endosomal sorting complexes required for transport (ESCRT) machinery mediates the invagination of the endosomal membrane and sorting of ubiquitinated cargo into intraluminal vesicles. MVBs eventually fuse with lysosomes, where the cargo is degraded.
