# Supplementary material for: Activation of the endocytosis pathway stratifies subtypes and therapeutic sensitivity in colorectal cancer
Source: Oncogene. 2026 Oct 19;45(46):5048–56. doi: 10.1038/s41388-026-04019-w (PMC13723413; doi:10.1038/s41388-026-04019-w)
Supplement: Supplementary file 2 [file 41388_2026_4019_MOESM2_ESM.pdf]

Figure S2

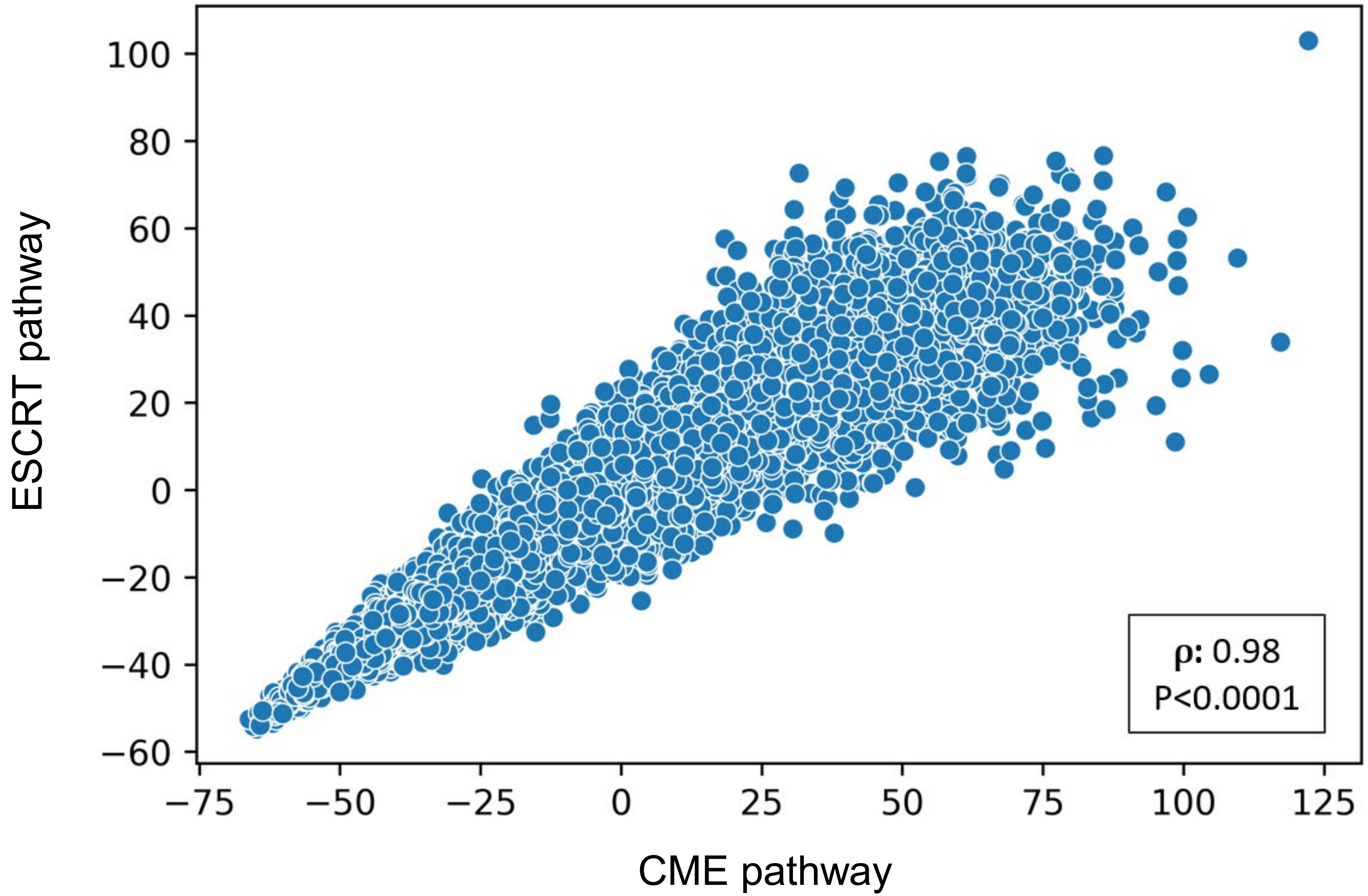

Z-scores of the CME and ESCRT pathways showed a strong positive correlation.
