# Supplementary material for: Activation of the endocytosis pathway stratifies subtypes and therapeutic sensitivity in colorectal cancer
Source: Oncogene. 2026 Oct 19;45(46):5048–56. doi: 10.1038/s41388-026-04019-w (PMC13723413; doi:10.1038/s41388-026-04019-w)
Supplement: Supplementary file 3 [file 41388_2026_4019_MOESM3_ESM.pdf]

**Figure S3**

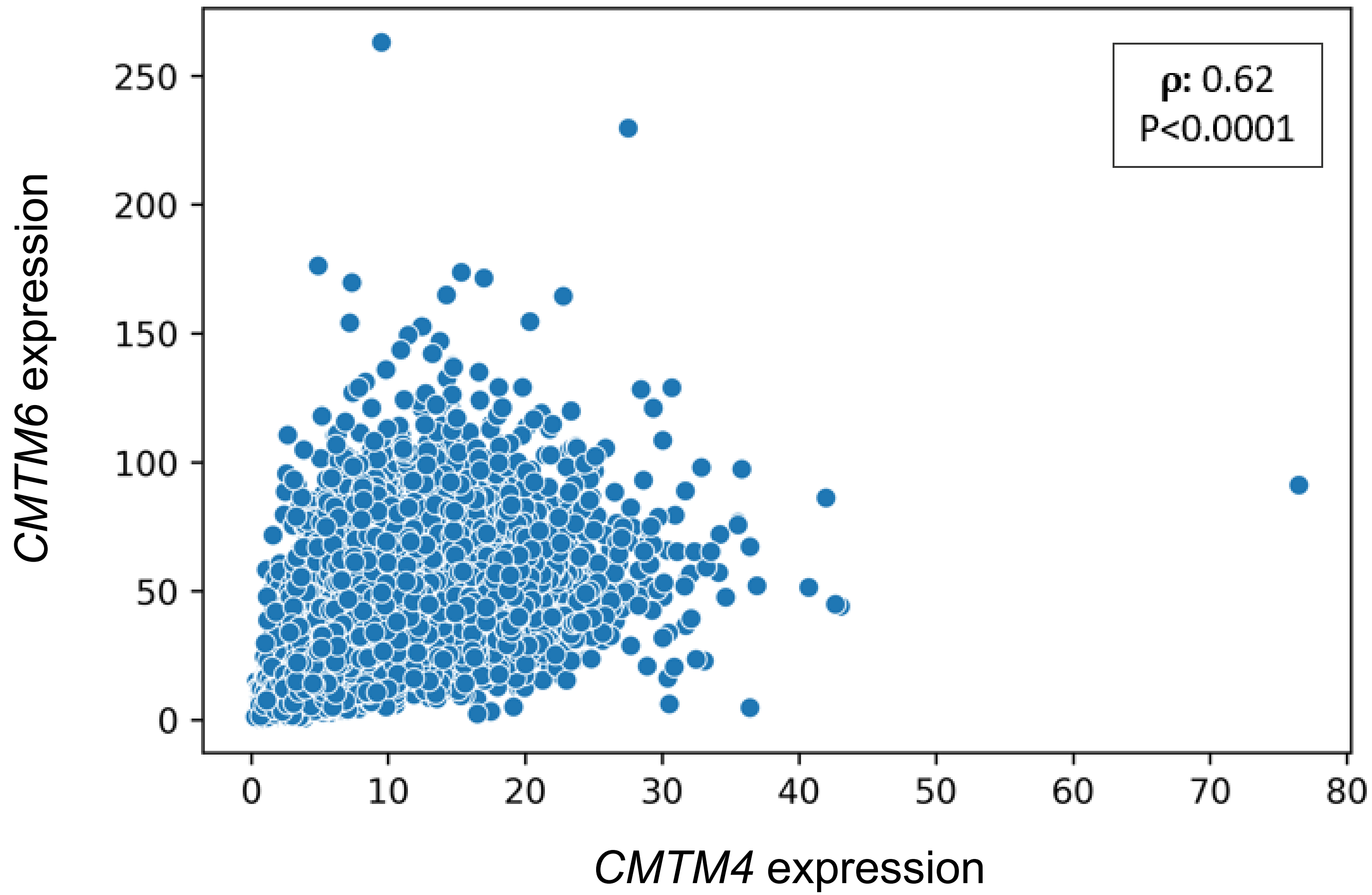


CMTM4 and CMTM6 are known to regulate PD-L1 recycling by preventing its lysosomal degradation, thereby sustaining PD-L1 surface expression. A strong positive correlation was observed between their transcript levels, suggesting coordinated regulation of this immune checkpoint–related axis.
