# Supplementary material for: Activation of the endocytosis pathway stratifies subtypes and therapeutic sensitivity in colorectal cancer
Source: Oncogene. 2026 Oct 19;45(46):5048–56. doi: 10.1038/s41388-026-04019-w (PMC13723413; doi:10.1038/s41388-026-04019-w)
Supplement: Supplementary file 5 [file 41388_2026_4019_MOESM5_ESM.pdf]

Figure S5

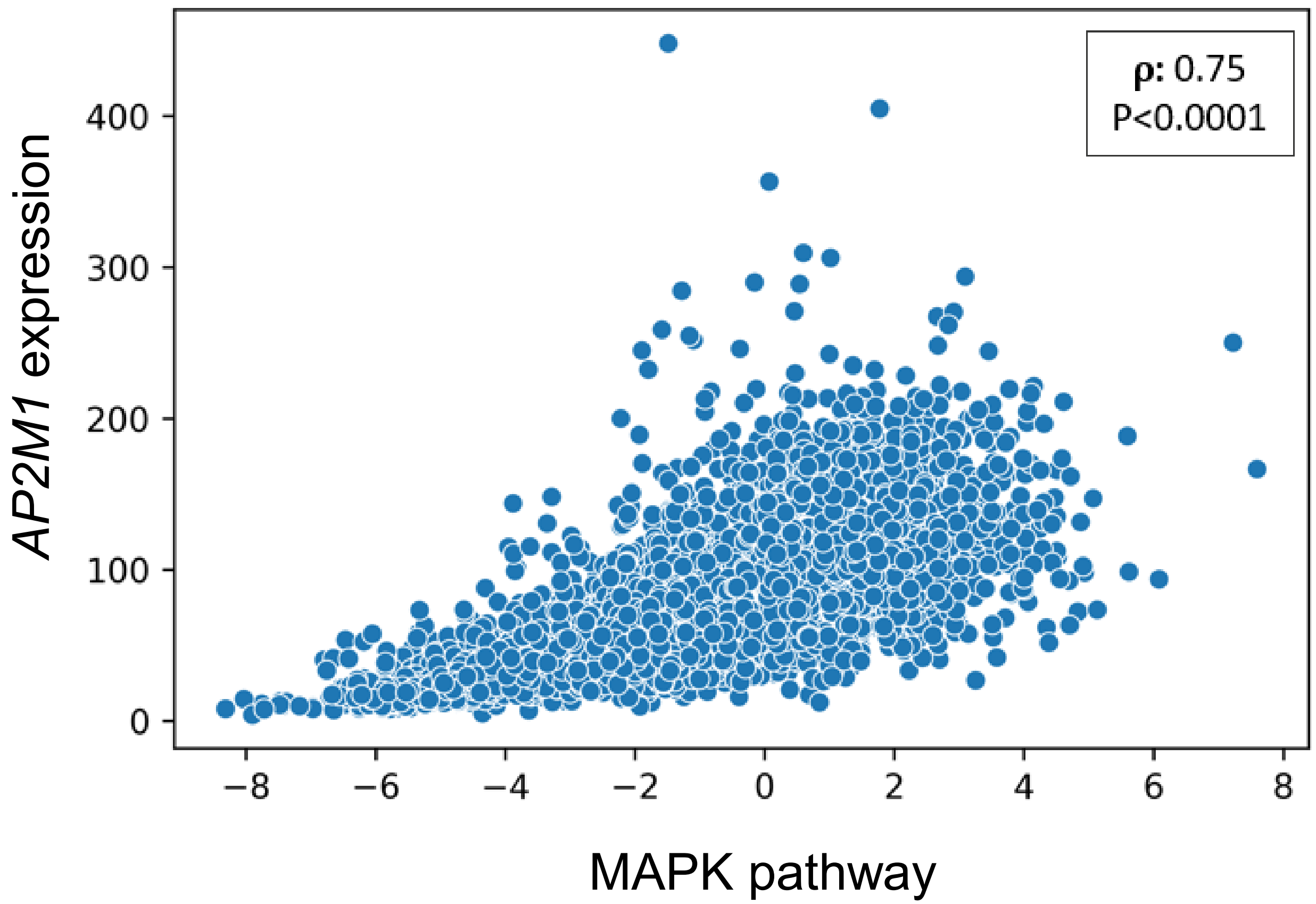

We analyzed the association between *AP2M1* expression and oncogenic signaling pathway activity in *RAS/BRAF* wild-type cases within the Caris cohort. The results were consistent with those observed in the overall cohort, showing strong correlations with multiple signaling pathways. Representative findings for the MAPK pathway are shown in the figure.
