# Supplementary material for: Activation of the endocytosis pathway stratifies subtypes and therapeutic sensitivity in colorectal cancer
Source: Oncogene. 2026 Oct 19;45(46):5048–56. doi: 10.1038/s41388-026-04019-w (PMC13723413; doi:10.1038/s41388-026-04019-w)
Supplement: Supplementary file 6 [file 41388_2026_4019_MOESM10_ESM.docx]

Table S4: Association between endocytosis-related gene expression and survival outcomes in cetuximab-treated patients (CALGB/SWOG 80405)

|  |  |  | PFS | | | | | |  | OS | | | | | |
| --- | --- | --- | --- | --- | --- | --- | --- | --- | --- | --- | --- | --- | --- | --- | --- |
|  |  |  |  | | Univariable Analysis | | Multivariable Analysis | |  |  | | Univariable Analysis | | Multivariable Analysis | |
| Gene | Group |  | Events/ Patients | Median Survival (95% CI)  (months) | HR (95% CI) | *P^*^* | HR (95% CI) | *P^*^* |  | Events/ Patients | Median Survival (95% CI)  (months) | HR (95% CI) | *P^*^* | HR (95% CI) | *P^*^* |
| *CLTC* | low |  | 94/99 | 11.3 (9.2, 14.2) | Reference | 0.46 | Reference | 0.10 |  | 79/99 | 32.4 (27.7, 39.2) | Reference | 0.59 | Reference | **0.03** |
|  | high |  | 105/108 | 10.7 (9.2, 13.0) | 1.11 (0.84, 1.47) |  | 1.30 (0.95, 1.77) |  |  | 90/108 | 26.7 (23.1, 35.7) | 1.09 (0.80, 1.47) |  | 1.46 (1.03, 2.06) |  |
| *AP2M1* | low |  | 100/106 | 13.3 (11.4, 17.5) | Reference | **0.00011** | Reference | **0.0011** |  | 78/106 | 39.4 (35.5, 50.6) | Reference | **4.4e-07** | Reference | **2.3e-06** |
|  | high |  | 99/101 | 8.9 (7.5, 10.9) | 1.74 (1.31, 2.31) |  | 1.73 (1.25, 2.39) |  |  | 91/101 | 20.9 (14.9, 26.0) | 2.17 (1.60, 2.95) |  | 2.30 (1.63, 3.25) |  |
| *GAK* | low |  | 96/99 | 10.9 (9.2, 13.2) | Reference | 0.52 | Reference | 0.42 |  | 81/99 | 28.9 (24.7, 40.8) | Reference | 0.77 | Reference | 0.35 |
|  | high |  | 103/108 | 11.0 (9.2, 13.4) | 1.10 (0.83, 1.45) |  | 1.14 (0.84, 1.54) |  |  | 88/108 | 32.4 (26.5, 35.8) | 0.96 (0.71, 1.29) |  | 0.85 (0.61, 1.19) |  |
| *TSG101* | low |  | 109/111 | 11.7 (9.3, 13.9) | Reference | 0.28 | Reference | 0.89 |  | 94/111 | 28.9 (24.9, 35.5) | Reference | 0.1 | Reference | 0.4 |
|  | high |  | 90/96 | 10.5 (9.2, 13.0) | 0.86 (0.65, 1.14) |  | 0.98 (0.70, 1.36) |  |  | 75/96 | 33.7 (26.0, 41.1) | 0.77 (0.57, 1.05) |  | 0.86 (0.61, 1.22) |  |
| *VPS37A* | low |  | 92/97 | 11.4 (9.8, 14.9) | Reference | 0.4 | Reference | 0.6 |  | 78/97 | 35.7 (30.5, 41.1) | Reference | 0.17 | Reference | 0.46 |
|  | high |  | 107/110 | 10.1 (8.1, 12.7) | 1.13 (0.85, 1.49) |  | 0.92 (0.67, 1.26) |  |  | 91/110 | 26.4 (21.7, 33.7) | 1.24 (0.91, 1.67) |  | 0.88 (0.62, 1.24) |  |
| *PDCD6IP* | low |  | 106/110 | 10.9 (8.7, 13.9) | Reference | 0.67 | Reference | 0.85 |  | 91/110 | 26.5 (21.2, 34.6) | Reference | 0.25 | Reference | 0.8 |
|  | high |  | 93/97 | 11.4 (9.6, 13.2) | 0.94 (0.71, 1.24) |  | 1.03 (0.74, 1.43) |  |  | 78/97 | 34.0 (28.9, 39.6) | 0.84 (0.62, 1.13) |  | 1.05 (0.74, 1.49) |  |
| *VPS4A* | low |  | 106/110 | 11.6 (9.8, 13.2) | Reference | 0.67 | Reference | 0.37 |  | 89/110 | 28.9 (24.7, 37.1) | Reference | 0.98 | Reference | 0.95 |
|  | high |  | 93/97 | 10.1 (8.7, 13.4) | 1.06 (0.80, 1.40) |  | 1.15 (0.85, 1.55) |  |  | 80/97 | 30.9 (26.7, 37.4) | 1.00 (0.74, 1.36) |  | 1.01 (0.73, 1.40) |  |
| *VPS4B* | low |  | 110/114 | 12.8 (10.9, 14.4) | Reference | 0.24 | Reference | 0.75 |  | 91/114 | 34.0 (30.0, 38.1) | Reference | 0.21 | Reference | 0.13 |
|  | high |  | 89/93 | 8.1 (6.2, 11.3) | 1.18 (0.89, 1.57) |  | 0.95 (0.69, 1.31) |  |  | 78/93 | 24.9 (15.9, 34.7) | 1.21 (0.89, 1.64) |  | 0.76 (0.53, 1.08) |  |
| ^*^*P* value was calculated comparing high vs. low gene expression. Log-rank test was used in univariable analysis and likelihood ratio test was used in multivariable Cox proportional hazards regression model adjusting for age, sex, ethnicity, ECOG performance status, tumor location, number of metastatic sites, *KRAS*, MSI status, and chemotherapy.  Abbreviations: CI, confidence interval; HR, hazard ratio; OS, overall survival; PFS, progression-free survival. | | | | | | | | | | | | | | | |
