# Supplementary material for: Activation of the endocytosis pathway stratifies subtypes and therapeutic sensitivity in colorectal cancer
Source: Oncogene. 2026 Oct 19;45(46):5048–56. doi: 10.1038/s41388-026-04019-w (PMC13723413; doi:10.1038/s41388-026-04019-w)
Supplement: Supplementary file 8 [file 41388_2026_4019_MOESM6_ESM.docx]

**Supplementary Methods**

**Next-generation sequencing (NGS) in the Caris dataset**

NGS was performed on genomic DNA isolated from FFPE samples using an NGS platform (Illumina, Inc., San Diego, CA). A custom-designed SureSelectXT assay (Agilent Technologies, Santa Clara, CA) was used to enrich 592 cancer-related whole-gene targets (Caris MI TumorSeek panel). All variants were detected with >99% confidence based on allele frequency and amplicon coverage, with an average sequencing coverage depth of 750 and an analytic sensitivity of 5%. Identified genetic variants were analyzed by board-certified molecular geneticists and categorized as follows according to the American College of Medical Genetics and Genomics standards: “pathogenic,” “presumed pathogenic,” “variant of unknown significance,” “presumed benign,” or “benign.” When assessing mutation frequencies of individual genes, “pathogenic” and “presumed pathogenic” were counted as mutations, whereas “variant of unknown significance,” “presumed benign,” and “benign” were excluded.

**Whole Transcriptome Sequencing (WTS) in the Caris dataset**

WTS uses a hybrid-capture method to pull down the full transcriptome from a FFPE tumor samples using the Agilent SureSelect Human All Exon V7 bait panel (Agilent Technologies, Santa Clara, CA) and the Illumina NovaSeq platform (Illumina, Inc., San Diego, CA). FFPE specimens underwent pathology review to diagnose percent tumor content and tumor size; a minimum of 10% of tumor content in the area for microdissection was required to enable enrichment and extraction of tumor-specific RNA. Qiagen RNA FFPE tissue extraction kit was used for extraction, and the RNA quality and quantity were determined using the Agilent TapeStation. Biotinylated RNA baits were hybridized to the synthesized and purified cDNA targets and the bait-target complexes were amplified in a post capture PCR reaction. The resultant libraries were quantified and normalized, and the pooled libraries were denatured, diluted and sequenced. Raw data was demultiplexed using the Illumina DRAGEN FFPE accelerator. FASTQ files were aligned with STAR aligner (Alex Dobin, release 2.7.4a github). A full 22,948-gene dataset of expression data was produced by the Salmon, which provides fast and bias-aware quantification of transcript expression.^[1]^ BAM files from STAR aligner were further processed for RNA variants using a custom detection pipeline. The reference genome used was GRCh37/hg19 and analytical validation of this test demonstrated ≥ 97% Positive Percent Agreement (PPA), ≥ 99% Negative Percent Agreement (NPA) and ≥ 99% Overall Percent Agreement (OPA) with a validated comparator method.

**Assessment microsatellite instability (MSI) and mismatch repair (MMR) status in the Caris dataset**

A combination of test platforms was used to determine the MSI or MMR status of the tumors profiled, including, IHC (MLH1, M1 antibody; MSH2, G2191129 antibody; MSH6, 44 anti-body; and PMS2, EPR3947 antibody [Ventana Medical Systems, Inc., Tucson, AZ, USA]) and NGS (for tumors tested with NextSeq platform, 7,000 target microsatellite loci were examined and compared to the reference genome hg19 from the University of California). The two platforms generated highly concordant results as previously reported ^[2]^ and in the rare cases of discordant results, the MSI or MMR status of the tumor was determined by IHC.

**Consensus molecular subtype classification**

Caris consensus molecular subtype (CMS) classifier was developed using RNA sequencing data collected from the WTS platform. A classifier was trained against the original CMS datasets (excluding a TCGA dataset of 512 cases) using a classic SVM model as implemented in R ^[3]^. A total of 600 genes were subsequently selected for each of the four CMS classifiers using One vs. Rest t-test to identify genes uniquely expression in each of the four classes. Cross-validation was performed to optimize the model and finalize SVM parameters. Possible overtraining was evaluated by predicting CMS subtypes from an independent blinded dataset (TCGA dataset of 512 samples) with an accuracy of 88.3%.

**Gene list for clathrin-mediated endocytosis (CME) and endosomal sorting complexes required for transport (ESCRT) pathways**

**CME-related genes (47 genes)**: *CLTA, CLTB, CLTC, FCHO1, FCHO2, AP2A1, AP2A2, AP2B1, AP2M1, AP2S1, EPS15, EPS15L1 (EPS15R), ITSN1, ITSN2, SNAP91, PICALM, EPN1, EPN2, AMPH (AMPH1), BIN1, SNX9, DNM1, DNM2, DNM3, DNAJC6, GAK, HSPA8, LDLRAP1, DAB2, STON1, STON2, AGFG1, NECAP1, NECAP2, NUMB, ARRB1, ARRB2, SYNJ1, SYNJ2, INPPL1 (SHIP2), OCRL (OCRL1), AAK1, SCYL2, DYRK1A, HIP1, HIP1R, CTTN*

**ESCRT-related genes (35 genes)**: *HGS (HRS), STAM (STAM1), STAM2, TSG101, VPS28, VPS37A (HCRP1), VPS37B, VPS37C, VPS37D, MVB12A, MVB12B, UBAP1, VPS25 (EAP20), SNF8 (EAP30), VPS36 (EAP45), PDCD6IP (ALIX), PTPN23 (HDPTP), BROX, CHMP1A, CHMP1B, CHMP2A, CHMP2B, CHMP3, CHMP4A, CHMP4B, CHMP4C, CHMP5, CHMP6, CHMP7, IST1, VPS4A, VPS4B, VTA1 (LIP5), USP8, MITD1*

**Gene list for six oncogenic signaling pathways**

**WNT pathway (HALLMARK_WNT_BETA_CATENIN_SIGNALING, GSEA mSigDB)**: *ADAM17, AXIN1, AXIN2, CCND2, CSNK1E, CTNNB1, CUL1, DKK1, DKK4, DLL1, DVL2, FRAT1, FZD1, FZD8, GNAI1, HDAC11, HDAC2, HDAC5, HEY1, HEY2, JAG1, JAG2, KAT2A, LEF1, MAML1, MYC, NCOR2, NCSTN, NKD1, NOTCH1, NOTCH4, NUMB, PPARD, PSEN2, PTCH1, RBPJ, SKP2, TCF7, TP53, WNT1, WNT5B, WNT6*

**MAPK pathway (MPAS signature, Wagle 2018)**: *PHLDA1, SPRY2, SPRY4, DUSP4, DUSP6, CCND1, EPHA2, EPHA4, ETV4, ETV5*

**PI3K/AKT/mTOR pathway (HALLMARK_PI3K_AKT_MTOR_SIGNALING)**: *ACACA, ACTR2, ACTR3, ADCY2, ADRBK1, AKT1, AKT1S1, AP2M1, ARF1, ARHGDIA, ARPC3, ATF1, CAB39, CAB39L, CALR, CAMK4, CDK1, CDK2, CDK4, CDKN1A, CDKN1B, CFL1, CLTC, CSNK2B, CXCR4, DAPP1, DDIT3, DUSP3, E2F1, ECSIT, EGFR, EIF4E, FASLG, FGF17, FGF22, FGF6, GNA14, GNGT1, GRB2, GSK3B, HRAS, HSP90B1, IL2RG, IL4, IRAK4, ITPR2, LCK, MAP2K3, MAP2K6, MAP3K7, MAPK1, MAPK10, MAPK8, MAPK9, MAPKAP1, MKNK1, MKNK2, MYD88, NCK1, NFKBIB, NGF, NOD1, PAK4, PDK1, PFN1, PIK3R3, PIKFYVE, PIN1, PITX2, PLA2G12A, PLCB1, PLCG1, PPP1CA, PPP2R1B, PRKAA2, PRKAG1, PRKAR2A, PRKCB, PTEN, PTPN11, RAC1, RAF1, RALB, RIPK1, RIT1, RPS6KA1, RPS6KA3, RPTOR, SFN, SLA, SLC2A1, SMAD2, SQSTM1, STAT2, TBK1, THEM4, TIAM1, TNFRSF1A, TRAF2, TRIB3, TSC2, UBE2D3, UBE2N, VAV3, TWHAB*

**Angiogenesis pathway (HALLMARK_ANGIOGENESIS)**: *AOPH, APP, CCND2, COL3A1, COL5A2, CXCL6, FGFR1, FSTL1, ITGAV, JAG1, JAG2, KCNJ8, LPL, LRPAP1, LUM, MSX1, NRP1, OLR1, PDGFA, PF4, PGLYRP1, POSTN, PRG2, PTK2, S100A4, SERPINA5, SLCO2A1, SPP1, STC1, THBD, TIMP1, TNFRSF21, VAV2, VCAN, VEGFA, VTN*

**TGF-β pathway (HALLMARK_TGF_BETA_SIGNALING, GSEA mSigDB)**: *ACVR1, APC, ARID4B, BCAR3, BMP2, BMPR1A, BMPR2, CDH1, CDK9, CDKN1C, CTNNB1, ENG, FKBP1A, FNTA, FURIN, HDAC1, HIPK2, ID1, ID2, ID3, IFNGR2, JUNB, KLF10, LEFTY2, LTBP2, MAP3K7, NCOR2, NOG, PMEPA1, PPM1A, PPP1CA, PPP1R15A, RAB31, RHOA, SERPINE1, SKI, SKIL, SLC20A1, SMAD1, SMAD3, SMAD6, SMAD7, SMURF1, SMURF2, SPTBN1, TGFB1, TGFBR1, TGIF1, THBS1, TJP1, TRIM33, UBE2D3, WWTR1, XIAP*

**NOTCH pathways (HALLMARK_NOTCH_SIGNALING, GSEA mSigDB)**: *APH1A, ARRB1, CCND1, CUL1, DLL1, DTX1, DTX2, DTX4, FBXW11, FZD1, FZD5, FZD7, HES1, HEYL, JAG1, KAT2A, LFNG, MAML2, NOTCH1, NOTCH2, NOTCH3, PPARD, PRKCA, PSEN2, PSENEN, RBX1, SAP30, SKP1, ST3GAL6, TCF7L2, WNT2, WNT5A*

**Reference**

1. Patro R, Duggal G, Love MI, et al. Salmon provides fast and bias-aware quantification of transcript expression. *Nat Methods.* 2017;14(4):417-9.
2. Vanderwalde A, Spetzler D, Xiao N, et al. Microsatellite instability status determined by next-generation sequencing and compared with PD-L1 and tumor mutational burden in 11,348 patients. *Cancer Med*. 2018;7(3):746-56.
3. Guinney J, Dienstmann R, Wang X, et al. The consensus molecular subtypes of colorectal cancer. *Nat Med*. 2015;21(11):1350-6.
