# Supplementary material for: Activation of the endocytosis pathway stratifies subtypes and therapeutic sensitivity in colorectal cancer
Source: Oncogene. 2026 Oct 19;45(46):5048–56. doi: 10.1038/s41388-026-04019-w (PMC13723413; doi:10.1038/s41388-026-04019-w)
Supplement: Supplementary file 9 [file 41388_2026_4019_MOESM7_ESM.docx]

**Table S1. Patient characteristics in the molecular profiling cohort (Caris dataset)**

|  | | *N* = 15025 | |
| --- | --- | --- | --- |
| Age | Median  (Range) | 62  (14->90) | |
| Sex | Male  Female | 8236  6789 | (54.8%)  (45.2%) |
| Primary tumor sidedness | Left  Right  Unknown | 8264  4632  2129 | (64.1%)  (35.9%)  - |
| Location of tumor sampling | Primary  Metastatic  Unknown | 8334  6380  311 | (56.6%)  (43.4%)  - |

Unknown groups were not included in the analysis.
