# Supplementary material for: Activation of the endocytosis pathway stratifies subtypes and therapeutic sensitivity in colorectal cancer
Source: Oncogene. 2026 Oct 19;45(46):5048–56. doi: 10.1038/s41388-026-04019-w (PMC13723413; doi:10.1038/s41388-026-04019-w)
Supplement: Supplementary file 10 [file 41388_2026_4019_MOESM8_ESM.docx]

**Table S2. Frequency of mutations and copy number alterations in 17 endocytosis-related genes (Caris MI TumorSeek Panel)**

| Gene | Mutation | Amplification |
| --- | --- | --- |
| *ARFRP1* | 0% | 1.03% |
| *SH3GL1* | 0% | 0.29% |
| *MDM2* | 0% | 0.08% |
| *PICALM* | 0% | 0.03% |
| *CLTCL1* | 0% | 0.02% |
| *COPB1* | 0% | 0.01% |
| *RABEP1* | 0% | 0.01% |
| *CLTC* | 0% | 0.01% |
| *HIP1* | 0% | 0.01% |
| *CBL* | 0% | 0.01% |
| *CBLC* | 0% | 0% |
| *CBLB* | 0% | 0% |
| *DNM2* | 0% | 0% |
| *EPS15* | 0% | 0% |
| *FNBP1* | 0% | 0% |
| *NDRG1* | 0% | 0% |
| *VTI1A* | 0% | 0% |
