# Supplementary material for: Activation of the endocytosis pathway stratifies subtypes and therapeutic sensitivity in colorectal cancer
Source: Oncogene. 2026 Oct 19;45(46):5048–56. doi: 10.1038/s41388-026-04019-w (PMC13723413; doi:10.1038/s41388-026-04019-w)
Supplement: Supplementary file 11 [file 41388_2026_4019_MOESM9_ESM.docx]

**Table S3. Patient characteristics in the clinical trial cohort (CALGB/SWOG 80405)**

|  | | CALGB/SWOG 80405 cohort | | | |
| --- | --- | --- | --- | --- | --- |
|  |  | Bevacizumab group  (*N* = 226) | | Cetuximab group  (*N* = 207) | |
| Age | Median  (Range) | 60  (24-80) | | 59  (30-83) | |
| Sex | Male  Female | 146  80 | (65%)  (35%) | 125  82 | (60%)  (40%) |
| ECOG PS | 0  1  2 | 131  95  0 | (58%)  (42%)  (0%) | 120  87  0 | (58%)  (42%)  (0%) |
| Primary tumor sidedness | Right  Left  Unknown | 95  128  3 | (42%)  (57%)  (1%) | 74  130  3 | (36%)  (63%)  (1%) |
| Primary tumor resection | Yes  No  Unknown | 214  9  3 | (95%)  (4%)  (1%) | 196  10  1 | (95%)  (5%)  (0.5%) |
| Number of metastatic sites | 1  ≥2  Unknown | 118  106  2 | (52%)  (47%)  (1%) | 104  102  1 | (50%)  (49%)  (0.5%) |
| *RAS* | Wild  Mutant  Unknown | 193  32  1 | (85%)  (14%)  (0.4%) | 172  35  0 | (83%)  (17%)  (0%) |
| *BRAF* | Wild  Mutant  Unknown | 175  37  14 | (77%)  (16%)  (6%) | 159  23  25 | (77%)  (11%)  (12%) |
| MSI status | MSI-H  MSI-L  MSS  Unknown | 18  9  181  18 | (8%)  (4%)  (80%)  (8%) | 11  10  151  35 | (5%)  (5%)  (73%)  (17%) |
| Backbone chemotherapy | FOLFOX  FOLFIRI | 174  52 | (77%)  (23%) | 149  58 | (72%)  (28%) |

Abbreviations: ECOG, Eastern Cooperative Oncology Group; MSI-H, microsatellite instability high; MSI-L, microsatellite instability low; MSS, microsatellite stable; PS, performance status.
